# Supplementary material for: Global distribution of isoprenoid quinones across Bacteria
Source: mSystems. 2026 Jul 1;11(7):e01681-25. doi: 10.1128/msystems.01681-25 (PMC13386864; doi:10.1128/msystems.01681-25)
Supplement: Dataset S1 — Optimized sequences of ubi gene candidates tested experimentally. [file msystems.01681-25-s0001.docx]

| >UbiX: GB_GCA_001595385.3 UbiX LVEI03000001.1_1442 - Optimized sequence：  CCATGGTGGCATATCTGATTGCACTGACCGGTGCAAGCGGTGCAATTTATGGTCTGCGTCTGGCGGGTGAACTGCTGAGTCGTGGTGATGATGTTGAAGTTATTATTAGTCCGAGTGGTTTTCTGATTCTGAAAGAAGAACTGGGTCTGGAATGTGCGCCGAAAGATGCAGCAAGCAAAATTCGTGCATATCTGGAAGGTCAGGGTCGTGCGCTGAAAGGCCGTCTGGGTATTACAGCACATGATGATATGAGCGCATCTGTTGCAAGCGGTAGTAGCCTGCTGAAAGCGATGATCATTTGCCCGTGTAGCATGGGTACCCTGGCACGTGTTGCAAGCGGCGTTAGTGGTAACCTGATTGAACGTGCAGCAGATTGTATGCTGAAAGAAAAGCGCCCGCTGCTGCTGGTTCCGCGTGAAACACCTCTGAGCAGCATTCATCTGCAGAATATGCTGCGTCTGTCACGTGCAGGTGCAGTGATTCTGCCGGCAATGCCGGCATTTTATCATAAACCTTCAACCATTGATGATATGGTGGATTTTATGGCAGGTAAAATTCTGGATATGCTGGGTGTTGAAAATAGCCTGTATAAACGTTGGAAGAAGGAAGCCGAATAAAAGCTT |
| --- |
| >UbiV: GB_GCA_001595385.3 UbiV LVEI03000001.1_1680 - Optimized sequence：  CATATGGAAATCACCCTGGGTCCGGTTCTGTTTGATTGGCCGAAAGATGAAGTTCTGAAATTTTATGAGGAGGCAAGTCGTATGGATGTGGATAGAGTGTATATTGGAGAGGTGGTGTGTACCCGGAAAATTGGTCTGCGTATGAATGACATTGAAGGTATAATTAAGCTGCTGCAGGATTCGGGAAAAAAAGTTATTCTGAGCACCCTGGCAGTTATTAGCAATGAAGAAGAACTGGAATTTACCCGTAAACTGCTGCATCTGCCGTGTCCGGTTGAAGCAAATGATATGAGCGTTTTTAATATGGCAGGTGAACGTGAACTGGTTGCAGGTCCGCATATTACCGCATATAATGCACCGACCATTGAATTTTTTAAAAGCATTGGTGTTAAACGTGTTGTTTTTCCGGTTGAACTGCCGAAAGCAAGCATTGAACATGATCTGCGTGCAACCGGTATTTTTGGTGAAGTTTTTGCACATGGTAAAGTTCCGCTGGCATTTAGCTGGCGTTGTTATACCAGCCGTGCATTTGGTCTGAATAAAACCAATTGTAAACATCATTGTATGAAATATCCGGATGGTATGGAACTGAAAACCGTTGATGGTGAACCGATTTTTAGCGTTAATGGTACCAGCATTCTGAGCGCAAGCACCTATAGCCTGGTTGAATTTGTTGAAGATCTGAAAGGTATTGGTGTTGGTGCACTGCGTATTAGCCCGCAGTATCGTAATACCGCAAAAATTGTTGAAGTTTTTCGTGCACGTGTTAATGGTACCCTGGGTCCGGGTGAAGGTATGAAAGAACTGAAAGCAGTTACCGAAGGTAGCTTTAGCAATGGTTGGTATCATGGTGGTGCAGGTAAAGAATATCTGAATGCAGTTCTGGGTTAAGGATCC |
| >UbiU: GB_GCA_001595385.3 UbiU LVEI03000003.1_59 - Optimized sequence：  CATATGACCAGACCAGAAGTTATTGCACCGGCCGGCAACCTGGCAAGCCTGAAAGCAGCAGTTGACAGCGGCGCCGACGCCGTTTACCTGGGCTTTAACGACGCAACCAATGCACGCAATTTTGAAGGCCTGAATTTTACCAGCAGCGAATTAGCAGAAGGAATCAAATACGTTCGGAGCAAAGGGAGACAGTTTTATGTTGCGATCAATACCTTCCCACAGGGCGAAGACTTTCCGAAGTGGTATAAAGCAGTTGACAGTGCAATGGAGATGAAAGCAGACGCAGTTATTATTGCAAACGTGGGAGTTTTACGGTATGCACGCCAGAAGTATCCAGATGCAACACTGCATCTGAGCACGCAGGCATCAAGTTCAAATTATGAAAGCATTAACTTCTACCGTAAGCACTTTGGTATTAAAAGAGTTGTTCTGCCCAGAGTTTTAACCATTGAAGAAATTAAACACCTGAAGGAACGCACAGAAGTTGAGATTGAAGTTTTTGCACTGGGTGGTCTGTGTATTAACATTGAGGGCCGCTGTTATTTATCCTCATACGTTACCGGAGCAAGCACCAACACCGAAGGTGCCTGTAGTCCGAGCCGTTTTGTTAGATTTAATAGTAATGACGACGGTGGTATGAGCATTGCACTGAATGGTATGACCCTTAATAAACTGGGCAAAGATGAAAGCAGCCCATATCCGACCTGTTGTAAAGGACGTTATACCGGTCCCGACGGCGGCTATGCATATATTTTTGAAGAGCCGGAATCGCTGAACGTTCTGGAGATCATTCCGGGTCTGATAGATGCAGGTGTTGCCGCACTGAAGATAGAAGGTCGTCAACGTACCAAAAGCTATGTTGGTGCCATGACCAGAGTTATGCGGGAAGCTGTGGATAGCTGTTGGAAGGATAGAGCAGGTTATACCGTTAGACCGGAATGGAGCAGCAAAACAGTGGCGACCTTTGAAGGAAGCCGTCAGACCCTGGGCAGCTATCTGACAAAATAAGGATCC |
| >UbiT: GB_GCA_001595385.3 UbiT LVEI03000001.1_1685 - Optimized sequence：  CATATGGAAGAAATCAAGAAGCAGCTGAGAGAAGAATTATATAAAGGACTGAGACTGCCGCTGAAAGCGATACCGCTGTGGATGGAAGCAATCGGGGTGGGGGTGTTTATAAAAAGCATCCTGGAAAAAAATCCGAGCTTTCGTGAACGTCTGGGTGAACTGGATGATAAAGTTTTTATGTTTGAAGCAAAAGATCTGGGTAAAGGTTTTTTTATGCATATTAAAGATAATGATATTAAAGTTAAACCGCATAGCGTTCGTGCACCGGATGTTACCATGAAAGGTGAAATGAGCGTTCTGATGGATGTTCTGCTGGGTAAAGAAGATCCGGATACCGTTTTTTTTAGCCGTAAACTGGAAATTACCGGTGATACCGCAACCGCAATTCATTTTAAAAATCTGCTGGCAGCACTGGGTTAAGGATCC |
| >UbiA: GB_GCA_001595385.3 UbiA LVEI03000001.1_1416 - Optimized sequence：  CCATGGTGGGTCAGGTTGCAGCAGATAAACTGCATGCAGTTAGTGAACTGCTGCGTCTGCCGCGTCAGCAGGGTACACTGCTGCTGCTGTGGCCTACAATGTGGAGTCTGTTTATGGCGAGTGGTGGTCGTCCGGAACTGAAATATCTGAGCATTTTCATTATCGGTGCTTTTCTGATGCGTTCAGCAGGTTGCGCCGTTAATGATATTGCAGATCGTGATTTTGATCCTCATGTGGAACGTACACGTACCCGTCCGATTGCTTCTGGTCGTCTGAAAGTTAAAGAAGCCATGCTGGTTTTCGCCCTGCTGAGCGCAGTTGCCTTTGCCCTGGTTCTGCAGCTGAATCGTCTGACCGTTATGCTGAGCCTGGTTGCCCTGGCCCTGGCAGGTGCATATCCATTTGTTAAACGTTTTAGTCATTTTCCTCAGGTTGTTCTGGGTATGGCATTTGGTTGGGGTGCAGTTATGGCGTGGTCCGCAGTTCGTGAAGAAGTTGGTGTTGCAGCACTGCTGATTTTCACCGCGAATATTTTCTGGAGTACTGCATATGATACTATTTATGCACTGATGGATCGTGATGATGATATTAAAATTGGTGTTAAAAGTACAGCCATCTTCTTCGGTGGTTCTGTTTATAAAGCACTGAGCGTTCTGTATCTGTGTTTTGCAGTTGCCCTGGGTGCCGCCGGTATGGTTGTTGGTCTGGGTGGTATTTTCATGACCGGCCTGCTGATTTGTCTGATTCTGAGCCTGGCCATTGTTGAATTTGTTAAGAAGGAACGTACCCGTCAGGCTGCGTTTAAAGGTTTTCAGGCTAATGCTGCCATTGGTGGTGTTCTGCTGCTGTTTATTATTCTGGATATGAATCTGTAAAAGCTT |
|  |
| >UbiC: GB_GCA_001595385.3 UbiC LVEI03000001.1_1415 - Optimized sequence：  CCATGGCTAAAGGCTTTAGCTATACCCTGCTGGGCCAGTGGCTGGGTGTTGAAGAAGCGCGTCGCAAAACAATCCTGGATGGTCTGCTGCCGCATCAGAAACTGCTGCTGTTTAGCGAAGGTAGCATGACCCTGGAACTGGAACTGCTGACCCAGGGTAATGTGGAAGCAGAAATTCGTTTTATGGGCCTGACTAGTATTACCGCCGAAGCAGCCTCATTTCTGGGTGCCGAAGTGGGTGCAGAAGCAATGGAACGTGAAGTGTGGCTGACCGGTGGCGGTCGTCGTCTGCTGTATGCACATGCACTGATTCCGGAAGGCATGATTGCACCGGATATTAAAAGTGCACTGGATGAACGTCCGAAAGAACCGCTGGGTCGTGTTCTGGCCAGTAATGGTGTTCTGTTTGCAAAAGAACGTCTGGAAATTGGTATTGTTAAAAGCCCGTGTGCAAGCCGTGATCTGGAAATTCCGGAAGATACCCCGCTGTTTGCTCGTCGTTATATTCTGTTTAATAAAGGTGCCGATCGTTGGATTATTAAAGCCGGTCTGACCGAAATTTTCAGTCCTGAACTGGTTGGTGCAGTTCTGCGTAGCTAAAAGCTT |
| >UbiD: GB_GCA_001595385.3 UbiD LVEI03000001.1_1417 - Optimized sequence：  ccatggcgccctactatgatctaagggaatttatagaggtcctggagaagaagggtttgctgaagcgcgttaaaaccgaagttgacccggttctcgaaatcgctgctattcaagaacgtctggtgaaaagcggtggtccggcggtgttgtttgaaaaagtgaaaggccaccgcatggcggttttgggaaacctgttcggcaccgcggagcgcgttgcgctgggcctgggcgtcaccgaagaagaattatcggatattggccagttcatagcgacgctgcaacgtccgcagccgcctgagggtctgtgggatgcggtgaagaagatcccgttcttcggtaagatcctgaccctgggtccgaagacggttaaatctgcaccgtgtcaggatgtcgtggagacagacacggcggatctgtccaaaattccgatcattaaatgctggccgggtgatgctgcgccactgatcacctggccattggttgtaactcaaagcccgcaaggtggtccgtataacgtgggcgtctatcgcatgcagtacctggatggtaagcgcgcgatcatgcgttggctgtcccatcgtggtggcgcaactcatcagcggctgtgggaaaaggagggcaaagcaatgccggtggcggtggccatcgggtgcgatccggcgacgatcatcgcgggcgtgacccctgtgccggaggacgttggtgagttccactttgcaggtgtattacgtaagaaggctatcgaattagttgagtgcaaaaccattccgttgaaggtgccggcgaccgcggaaattatcattgagggtgagatccgtcacggcgaactggaaatggaaggcccgtttggtgaccataccggttattacaacgctgctgaaccgttcccggtatttcatgttaaagccatcacccaccgtaaagatgccatctatatgaccaccattaccggccgtccgccgaaagaggacgctgtgatcggcacggttttgaataagctgtaccttccgagcctgaagctgcaatttccggaagttgttgatttttgtctgccgatggaagcagtttcctaccgcattgccgtggtgtccattaaaaaggagtacccgggccacgcaagacgtattatgatgggcctgtggggtgttttgaagcagttcatgtatgttaaatacattatcgtggtggacgacgacgtggacgtccacaactggaccgatgtcatctgggcaatcagcacccgtgttgaccccaaacgtgataccttgatcattgagaacacgccgatcgattatctggacttcagcagcccgattgagaatctaggtagcaaaatgggtattgacgcgaccaataagtacccgccagaggtgagccgtaaatggggtgagaaaatggaaatggacagcaaagttgaggaactcgtagagaagaagtggaaagagtacggcttttaaaagctt |

>UbiG: GB_GCA_001595385.3 UbiG LVEI03000001.1_1418 - Optimized sequence：

Ccatggcgactacagagtcacagaaatttgaacaatatggaagtgattggtggaatccggcaggccgtctgttctccttacatcgtattaacccactgcgcttcggttacttctccagccgtagcggcgaactggcgggcaagaccgttttggacatcggctgcggtggtggcctgctgagcgaggagttcgccaaggcgggcgcaaccgtcaccggtattgacctgtctccggttgcgattgacgctgcgaaaggtcactgcgcggcgtctggcttgtcgatcgactaccgcgtggcctccgtggagaaaacggctcgtgaaggtaaacaatttgatgttattgtctgtgcggaagttctggagcacgttgatgacttaaatggttttcttagagacagcctgtctatgctgaagcacggcggtctgttctttttcggcaccatcaacaaaacgtttaaggctcgtttcctcgctctgtttatggcagaagacgtgttgggtatggttccgcgtggtactcatgattataaccgtttcgtgcgtccgagcaccctgaaagaaatcctagcgcagaacggggtggagatcgaagagctgaagggtatgagctatgatccgttgcgcttggagtttaagatcagcaatgataccagcgtgaactatctgggctacgcacgcaaaaaataaaagctt

>UbiB: GB_GCA_001595385.3 UbiB LVEI03000001.1_1682 - Optimized sequence：

ccatggcggcttcagcatataaaaatataagaaggctaaaccgtatcgttattaccctgatccgctacggctttggtggcttagcacgtgacctgcgtgtactgccgagttttgttccggcaatcgagcgattgttcatctccaagaaagcgcgtgatttatccgccccggtgcgtattcgtctagtgcttgaagagctgggtccgaccttcattaagctgggtcagatcgccagcacccgtgcagatatcctgccaccggattgggtggaggaattcaagaagctgcaagatatggttccgccggttgagttcgaggaagttcgtcgtattatcgagggcagcttcaaggcgcctattggggcgaagttcgcctcgttcgacaccgaaccggtggcgtctgcgagcatcgcgcaggttcattatgcggaactgttcgacggctccaaggtggccgttaaagttcgccgtccaggtatcgagcgcgtcattgcgtccgacattagcgtcatgcacaccattgccggtctactggatcgctacgtgagtgctgctcgtcgctaccgtccgcacgaagttgtttctgaattcgagcgcgtgatcaagtccgagcaggatttgacgattgagggcgtgaacttgaatcgtttcagcgacattttcaaagacgacccgcacatccagatcccgcgtgtgttctgggattacaccactgaggacgtgctgacgatggagcgtatttctggcaccccgatcgacgaagtcgaaactctgaaaagcaagggtattgacatcaaggaggttgcggttcgcggcattggcattttctttaagcaggtttttgagcacggcatctttcacgccgacctgcatccgggtaacattttcgtgcgtgatgatggtgttatcatttatctggattttggtatcatcggccgtctggaccgtgatctgcgtaagtatctggcgagcatgctgtttcatctggtgcgcagcgattattaccgcatggctttagtgcaccgtgaaatgggtttgatcggcgatgatgtttccctgagcgagttcgaagaagcgttgcgggacattagcgtaccgatctttggtaaatcgctggagaaaatcgacatcagcggtctgctgatgaaactcttacagaccgcgaaacgttttaacatgaaattgcaaccgaatttgctcctgttacaaaaaagcatggtgataatcgagggtgtcggtcgtcaattgtacccggacgtgaacatgtgggaagtggcaaaaccactgattttcaaatggatggctaaagaaaagctgtctccgaaaatgatcgcggaacgtggccgcgaaaaactggaggaaattatggaaacggcgtttgacttgccggtcaattttaacaccctgctgcgcagaacgctgcgcgaggacatcaagatcggcttcgtgcatcatagactcgaagaggtcaccaatgagctggagaatgcaggccgtcgtattggtggcggtatcgtggttgcggcactggtcattggtgcgtcgctggtggcagttttcagcaaagaggcgaccacgtttctgggcctcccggttttgagcggcgctggttatttggttgcagcgatcatgggtctgcgtttgtttcgtcgtagcggacgcaacggtaggtaaaagctt

>UbiE: GB_GCA_001595385.3 UbiE LVEI03000001.1_1684 - Optimized sequence：

Ccatggcgtcagaaaaaatgacacactttggaaatcaaactatcccggaaggtgagaaagagaagaaggtgcgtgaagtttttgacagcgttgctagccgttacgacctgatgaatgatttgatgagcttcggtgttcaccgtttctggaaacgttttgttgcggcggaaaccggcctgcgtccgggccaaagcgcgattgatgttgccggtggaaccgcagacatcagcctgctgatggcagaccgcgtgggcgaggccggcaacatcgttgtgtttgacatcaacggtgagatgctgaagtatggtaaagagaaatgtgttgatcgcggttacttgaagaacatccgcttcgtgcagggtaatgcggaagatattgcgttcgacgacaacaccttccactgcgctaccgttggttttggcattcgcaacgtgacgcatctggatcgtgcttttcgtgagatgacccgtgtcgtgaaaccgggtggtaaagtcatctgcctggaattttcccatccgacgagcaaactgttcaaaaaggcgtacgatttatattcgttctcttttattccgaatgtaggcgagatgattaccggtaatcgctctgcgtatgaataccttccggagtccatccgtaaattcccaccgcaggaggaattgaagaagattatggaaggcgcaggtctgtggaaagtgaagtaccataacctcatgaacggcatcgccgcggttcacgtgggcgtcaaggtgtaaaagctt

>RquA: GB_GCA_001595385.3 RquA2 LVEI03000001.1_991 - Optimized sequence：

ccatggacatatatgaattacaagattcaaggcccctagctgaggaacgctggctgcggaagggactgttttatcgttactttctggacggcgtgccggattacctggctcgtaactattggtgggcctacttatggaaaccgggcgcatggtttttcgaccaccagccgattatcaatgcgatcctgttcggccaataccagcgtcttatgggtgaaacgctgcgcgtgatccaagcaagacctagcggtcgtatgctgcaactgtcttgcgtttacggcaagctgaccccgagtttggcgggtctggactctcgtcgtttgcacctgaccgatgtgtccccggtgcagctgggcattagcatgcgtaaggcgggcgagaaactggtggccacccgtatgaacgcggagagcttgggttatcgtgacggtgttttcgacaccgttctcatttttttcctgatgcatgaaatgccgccagaagctcgtcgccgtactctcagcgaagccatccgcgtactgtcgccgaagggccgtttggtcatcaccgaatatggtcatgagccgaatgcgaacccgatctatcgctttcgtttgtccagatggattattggtaagttggaaccttttctgccgggtttctggcgtgaggaactggatgttagcatgaaaaacgcggcaagcattaatcgcaagacgatcaaaaggaacggtaaagatgttccggttttcaaaggcttctaccgtgtcgcggagtacgaggtggagtaaaagctt
