# Supplementary material for: Global distribution of isoprenoid quinones across Bacteria
Source: mSystems. 2026 Jul 1;11(7):e01681-25. doi: 10.1128/msystems.01681-25 (PMC13386864; doi:10.1128/msystems.01681-25)
Supplement: Supplemental Material — Fig. S1 to S8, Texts S1 to S3, and captions for supplemental tables and data sets. [file msystems.01681-25-s0002.pdf]

Supplementary material for:

Global distribution of isoprenoid quinones across Bacteria

Sophie-Carole Chobert[1,†], Suraj Kanwar[1], Olivier Lerouxel[1], Nelle Varoquaux[1], Julie Michaud[1], Ludovic Pelosi[1], Fabien Pierrel[1]*, Sophie S. Abby[1]*

[1] TIMC, UMR 5525, VetAgro Sup, Grenoble INP, CNRS, Université Grenoble Alpes, Grenoble, France

[†] Present address: Univ. Grenoble Alpes, CNRS, INRAE, IRD, Grenoble INP, IGE, 38000 Grenoble, France
* Corresponding authors

Supplementary figures:

Figure S1. Chemical structure of isoprenoid quinones.
Figure S2. The two menaquinone pathways.
Figure S3. Phylogenetic tree of bacteria with a detailed view of quinone pathways.
Figure S4. Phylogenetic tree of « Deltaproteobacteria ».
Figure S5. Phylogenies of UQ genes: UbiA, UbiB, UbiC, UbiD, UbiE and their homologs in PQ and mPQ biosynthetic pathways.
Figure S6. Phylogenies of UQ genes: UbiU and UbiV.
Figure S7. Distribution of chain length variation in Actinomycetes.
Figure S8. Text-mining data analysis.

Supplementary texts:

Text S1. On the cases of discordance between the genomic annotation and the text-mining data.
Text S2. On the variability of quinone chains.
Text S3. On the species mentioned multiple times in the text-mining data.

Supplementary tables:

Table S1. Genomic annotation of quinone biosynthetic pathways
Table S2. Text-mining results
Table S3. Genomes selected for the species trees
Table S4. Plasmids used in this study
Table S5. *E. coli* strains used in this study
Table S6. Cases of discrepancy in species mentioned multiple times

Supplementary dataset:

Dataset S1. Optimized sequences of *ubi* gene candidates tested experimentally

Dataset S2. Phylogenies and supporting data for phylogenies (available on Figshare: 10.6084/m9.figshare.30145930)

Supplementary figures

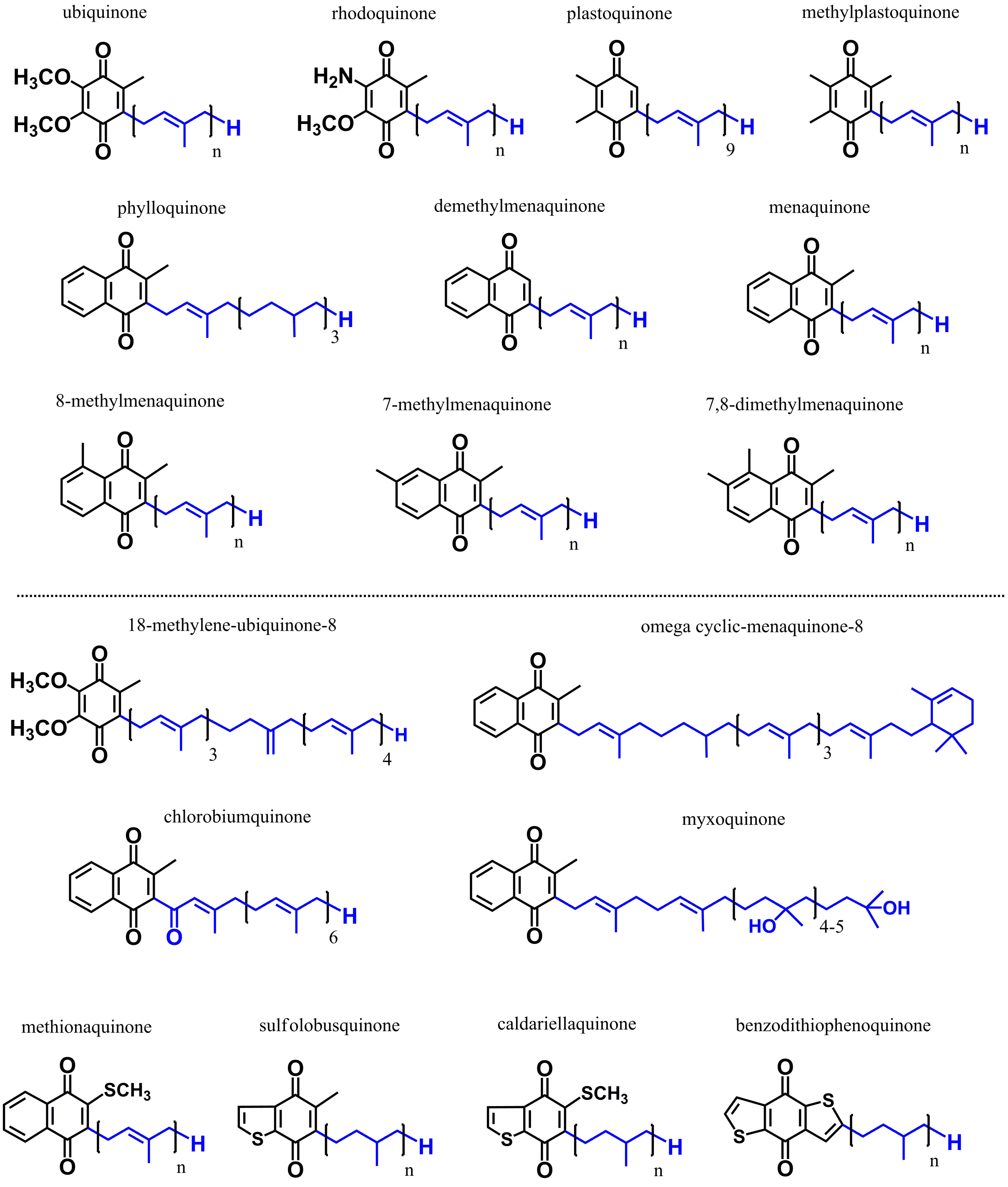

Figure S1. Chemical structures of isoprenoid quinones. The biosynthetic pathways of quinones represented beneath the dashed line remain uncharacterized.

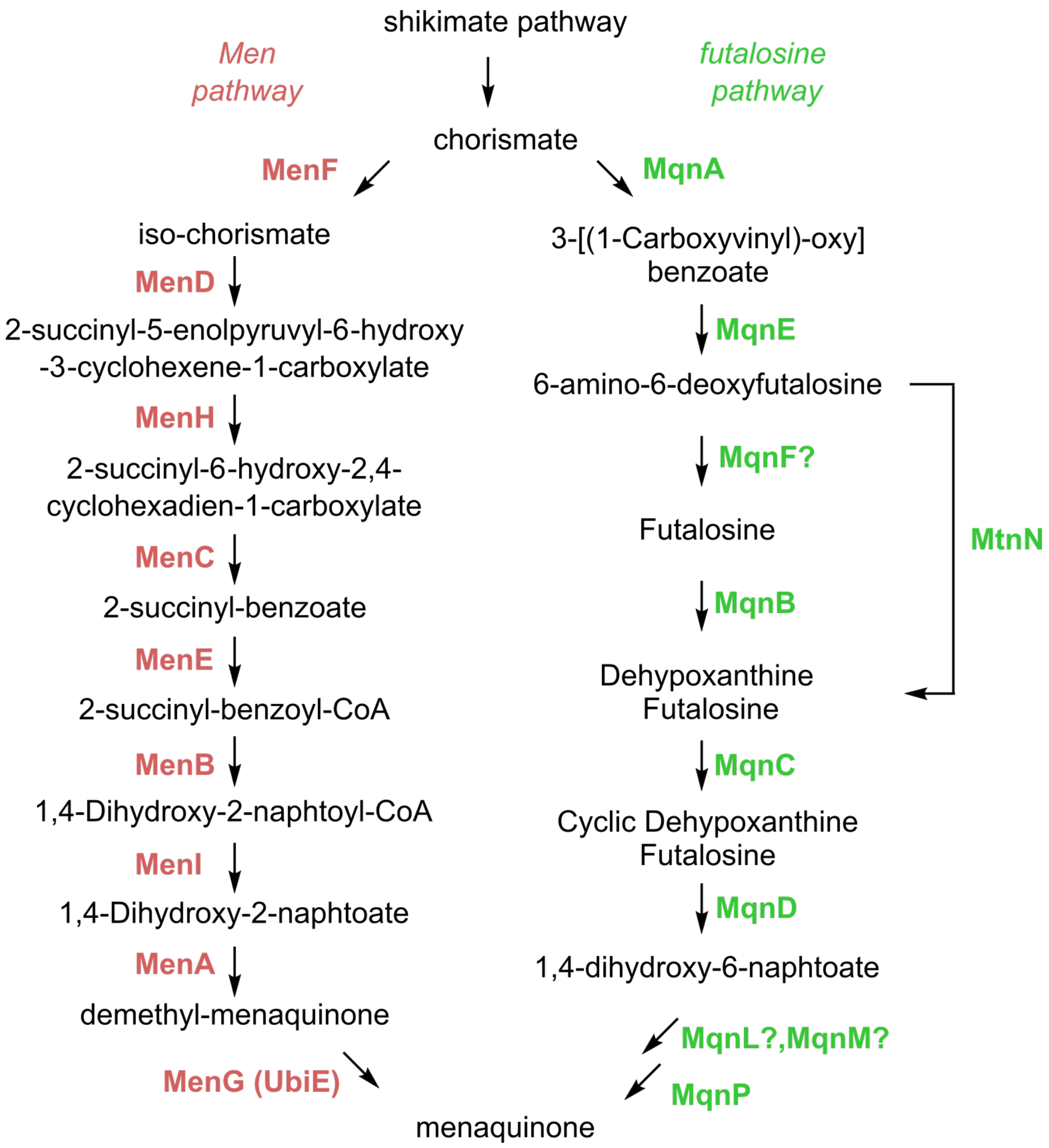

Figure S2. Menaquinone production by the Men and futalosine pathways. Modified from (1).

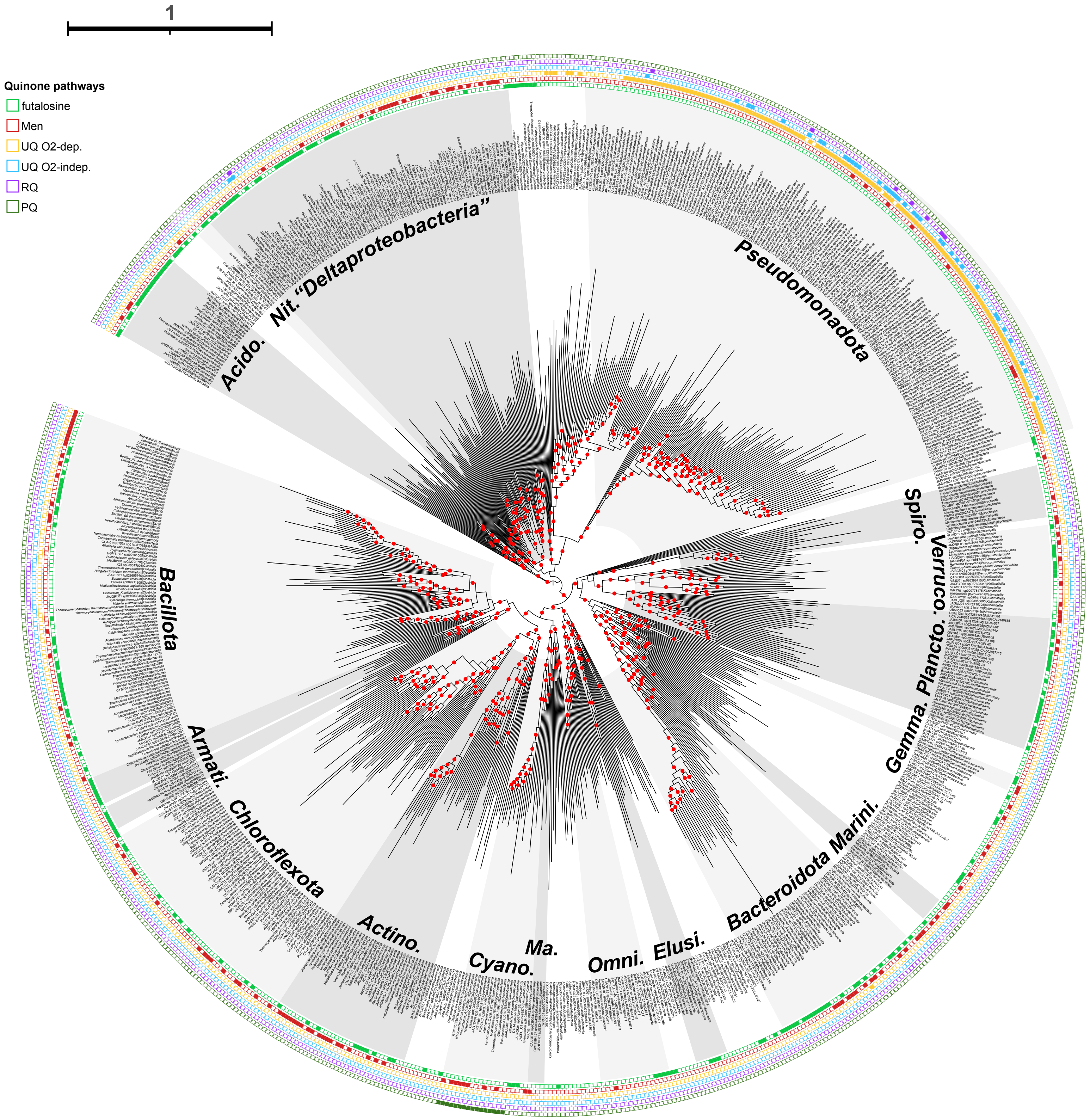

Figure S3. Phylogenetic tree of bacteria with a detailed view of quinone pathways. Tree presented Fig. 1. Bootstrap values over 95% are depicted by red circles. The tree was rooted to separate the Gracilicutes from the Terrabacteria. The following selected phyla are labeled on the tree: *Pseudomonadota*, *Spirochaetota*, *Verrucomicrobiota*, *Planctomycetota*, *Gemmatimonadota*, *Marinisomatota*, *Bacteroidota*, *Elusimicrobiota*, *Omnitrophota*, *Margulisbacteria*, *Cyanobacteriota*, *Actinomycetota*, *Chloroflexota*, *Armatimonadota*, *Bacillota*, *Acidobacteriota*, *Nitrospirota* and “Deltaproteobacteria” (*Desulfobacterota*, *Myxoccocota*, *Bdellovibrionota* and relatives). The prediction or absence of biosynthetic pathways—MK futalosine (green), MK Men (red), UQ O_2-dependent (orange), UQ O_2-independent (blue), RQ (purple), and PQ (dark green)—is specified along the tree.

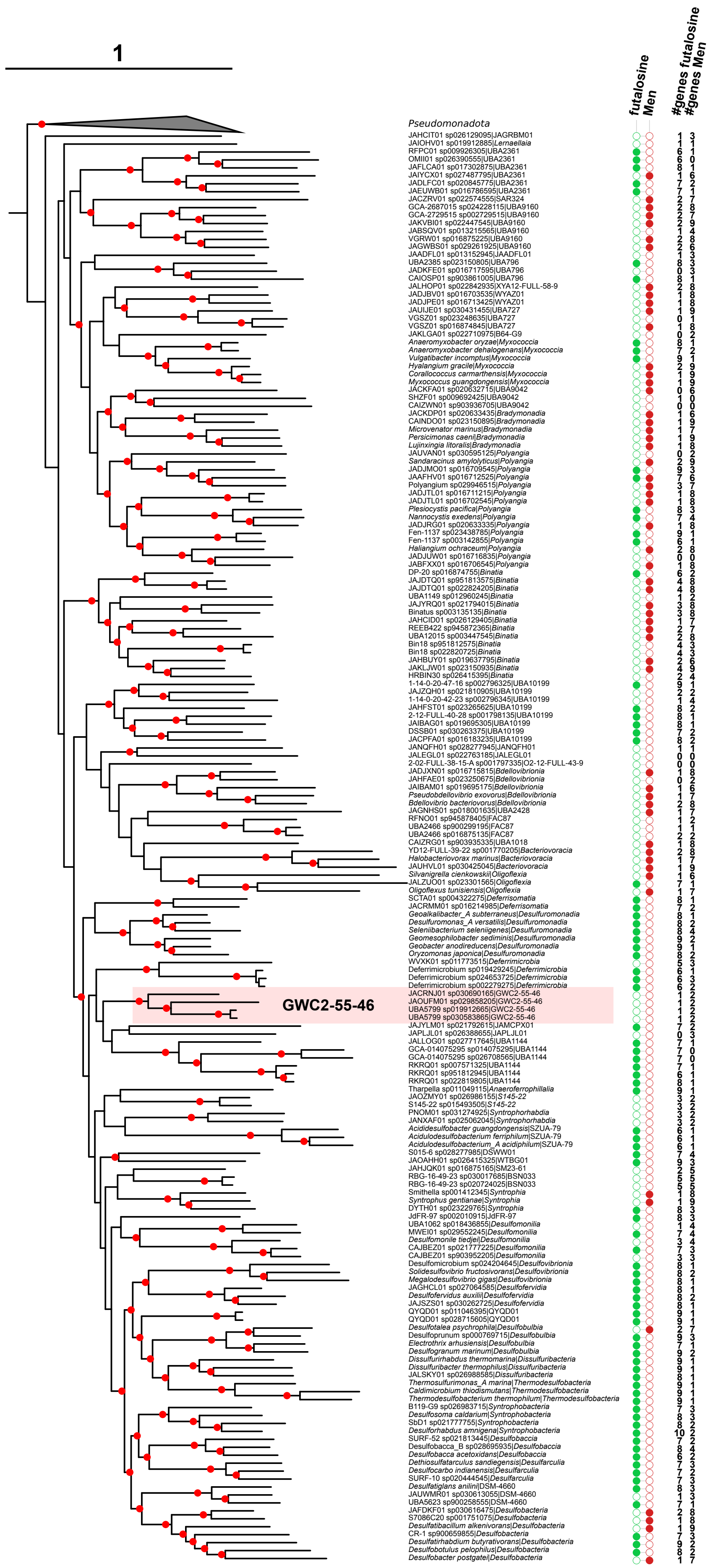

Figure S4. Phylogenetic tree of « Deltaproteobacteria ». Tree presented in Figure 2C. The prediction or absence of biosynthetic pathways—MK futalosine (green), MK Men (red)—is specified along the tree as well as the number of genes identified for each of the two pathways.

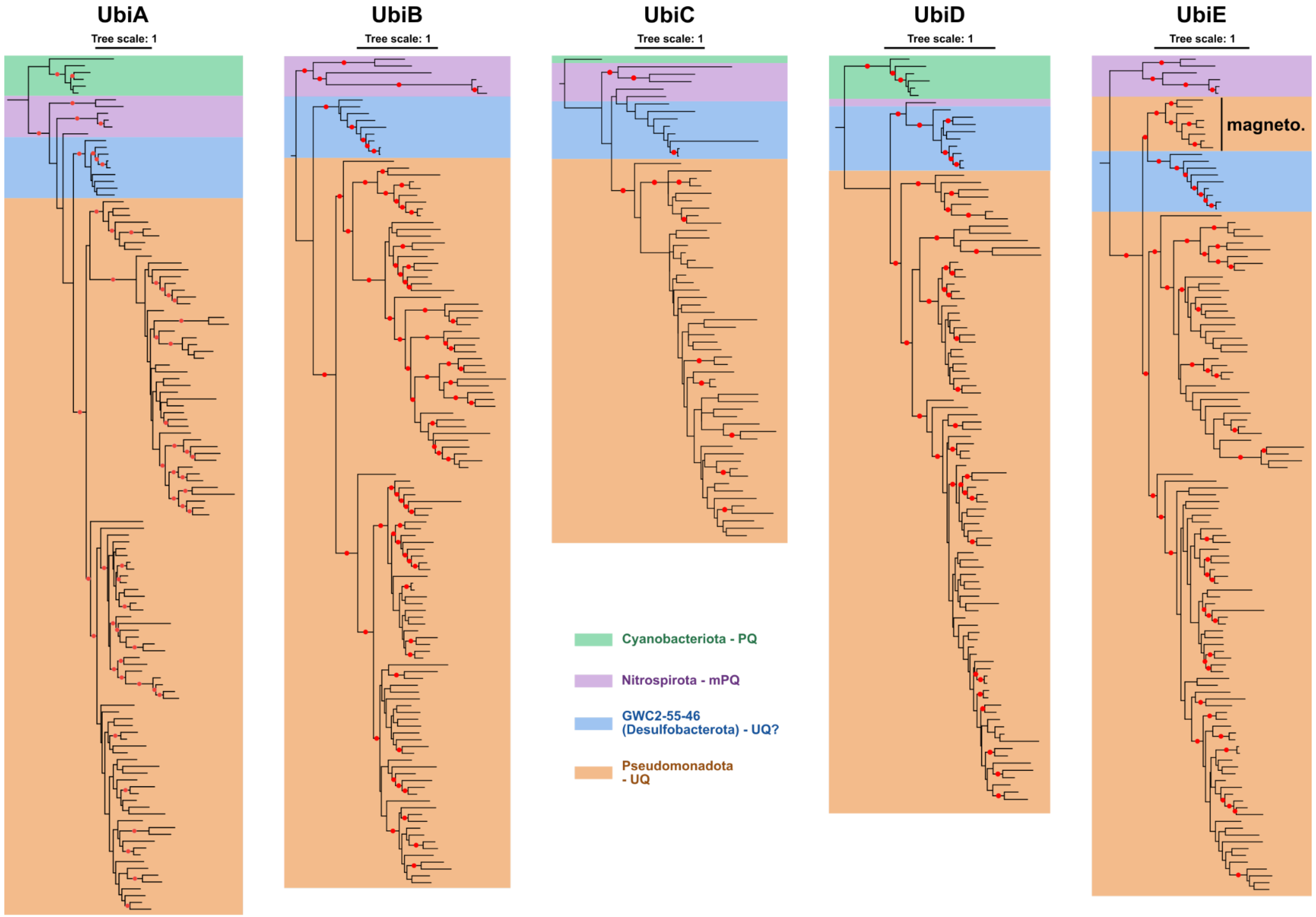

Figure S5. Phylogenies of UQ biosynthetic proteins: UbiA, UbiB, UbiC, UbiD, UbiE and their homologs in PQ and mPQ biosynthetic pathways. The trees of UbiA, UbiB, UbiC, UbiD and UbiE were obtained from the analysis of 247, 422, 79, 474 and 233 aligned positions respectively using IQ-TREE with LG+F+I+I+R7 as the best selected model for UbiA, LG+F+R7 for UbiB, Q.pfam+I+G4 for UbiC, LG+R6 for UbiD, Q.pfam+I+I+R7 for UbiE. The tree scale bar expresses the number of substitutions per site. The branches with very high support (UFBoot ≥ 95%) are indicated by red dots. The trees are rooted using *Cyanobacteria* or *Nitrospirota* sequences as an outgroup when no homologs are found in Cyanobacteria. Overall, tree topologies are close to one another. In the UbiE tree, the group formed by sequences from GWC2-55-46 is positioned as a sister group to a class of *Pseudomonadota*, the *Magnetococcia*.

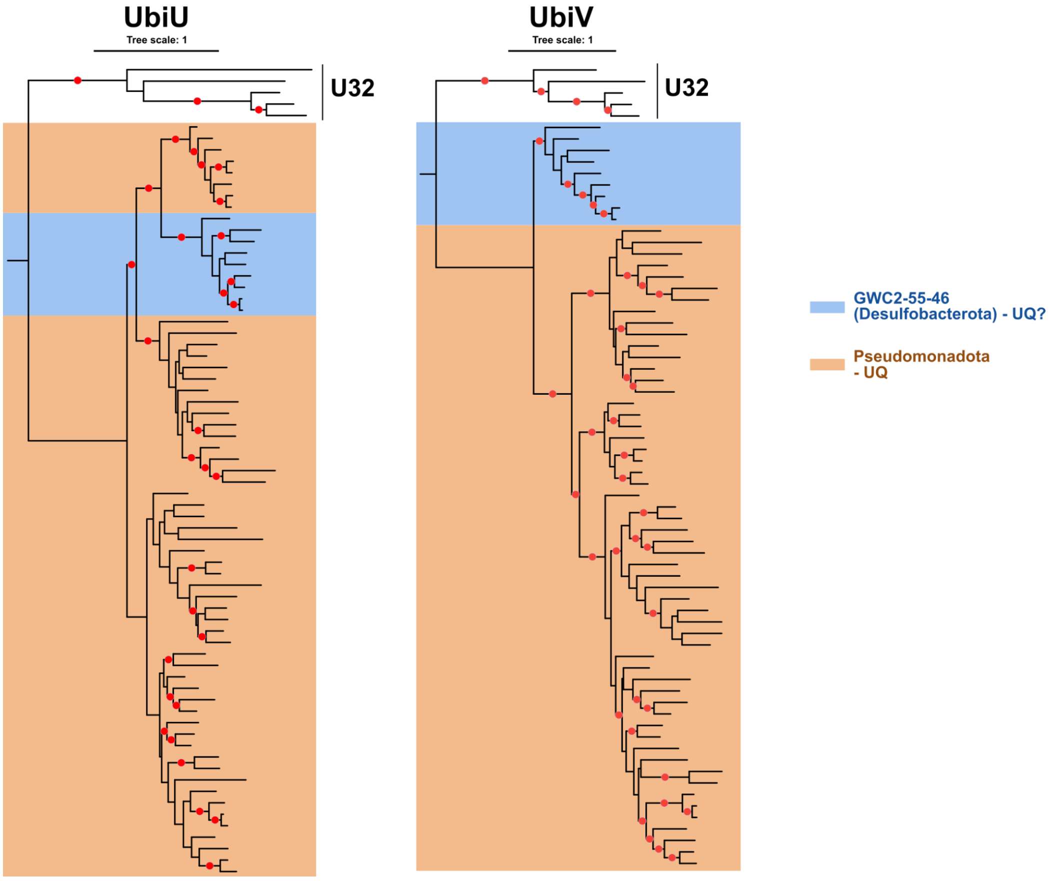

Figure S6. Phylogenies of UQ biosynthetic proteins: UbiU and UbiV. The trees were obtained from the analysis of 302 and 251 aligned positions for UbiU and UbiV using IQ-TREE with Q.pfam+I+G4 as the best selected model for both of the trees. The tree scale bar expresses the number of substitutions per site. The branches with very high support (UFBoot ≥ 95%) are indicated by red dots. U32 protease sequences were used as an outgroup.

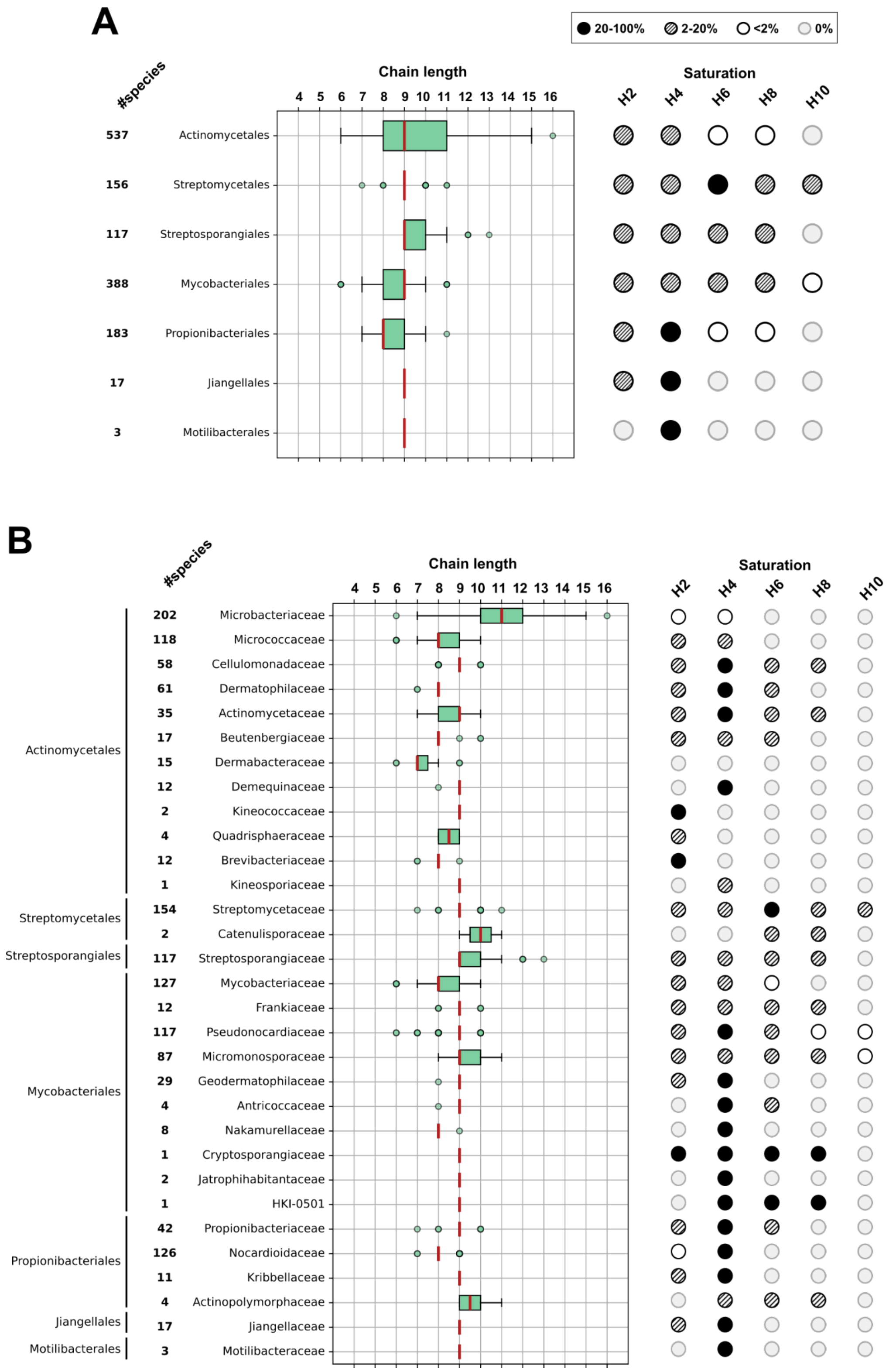

Figure S7. Distribution of chain length variation in Actinomycetes. Chain length variation and saturations at order **(A)** and family level **(B)**. The boxplots follow the typical rule: the median (Q2) is shown in red, 50% of the data is covered by the box (Q1, Q3), and the ends of the whiskers correspond to the minimum and maximum chain lengths within 1.5 times the interquartile range before Q1 and after Q3. Outliers are represented by small circles. Circle edge is thick when two or more outliers share the same value. The number of unique species per order/family is specified along the boxplots.

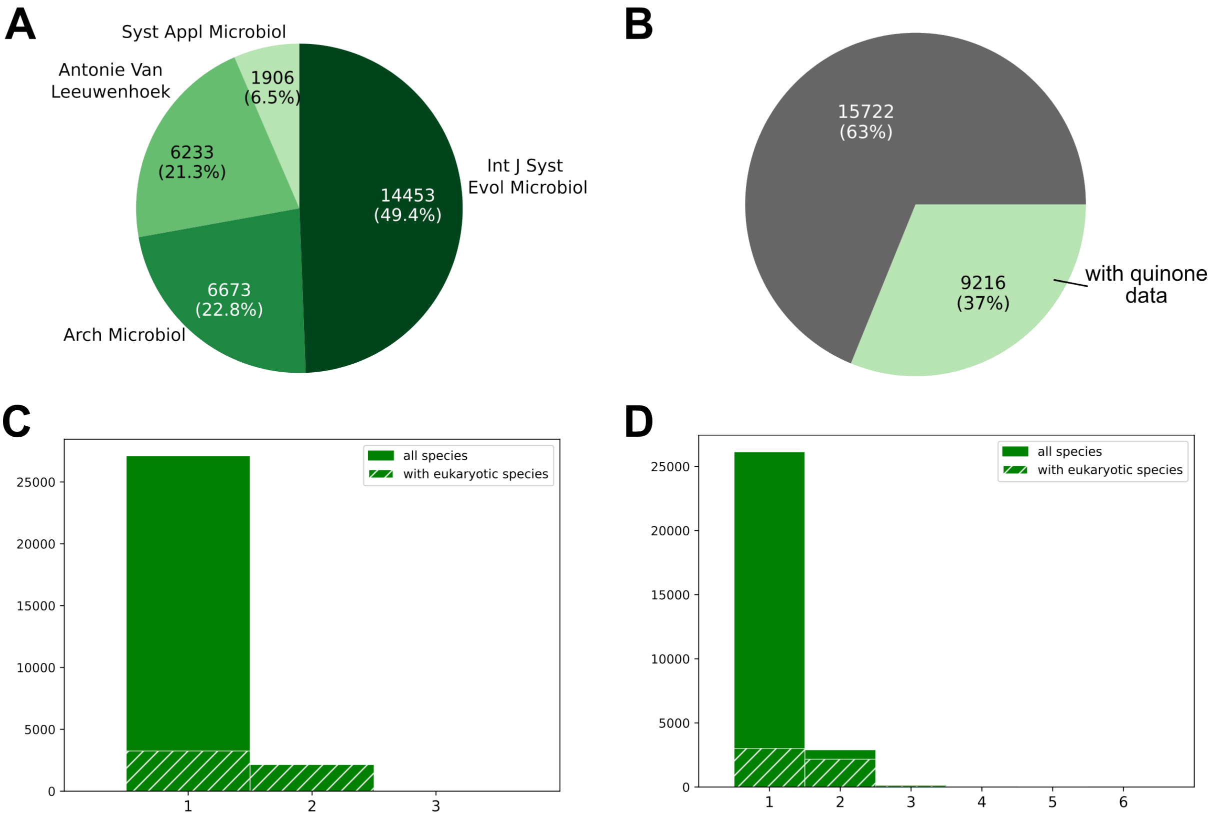

Figure S8. Text-mining data analysis. **(A)** Proportion of abstracts from each journal: Int J System (*International journal of systematic and evolutionary microbiology*), Arch Microbiol (*Archives of microbiology*), *Antonie Van Leeuwenhoek*, Syst Appl Microbiol (*International journal of systematic bacteriology*) **(B)** Proportion of articles from which information on the type of quinone associated with prokaryotic taxonomic information could be extracted. **(C)** Distribution of the number of different divisions (NCBI taxonomy) per article. The proportion of articles where at least one of the two species is a eukaryote is indicated by hatching. **(D)** Same as C but at genus level. In the context of these articles specialized on prokaryotes, the mention of a eukaryotic species mostly refers to the host species of the bacterium in cases of symbiosis or the source of isolation. In the vast majority of cases, when two genera are mentioned, the other species/genus is a eukaryote. We therefore assume that the description of the quinone refers to the prokaryotic species mentioned.

Supplementary texts

Text S1

A few notable cases of discordance between the genomic annotations and the text-mining data were identified (Fig. 1, Tables S1 and S2): MK was measured in *Romboutsia ilealis* (*Bacillota,* PMID: 24480908), *Thermacetogenium phaeum* (*Bacillota_B*, PMID: 10939667), *Thiogranum longum* (*Pseudomonadota,* PMID: 25336721) and *Mesosutterella multiformis* (*Pseudomonadota*, PMID: 30394865), whereas we did not detect any MK pathway in the genomes of these species. In the first three species, no more than two genes involved in MK production were found, suggesting that the genetic potential for MK production is largely incomplete. In contrast, *M. multiformis* harbors five MK-related genes, just below the threshold of six genes required to infer the presence of the MK pathway. In *T. longum,* UQ is annotated, as expected for a *Pseudomonadota*. However, only MK_8-H_4 and MK_9-H_4 have been observed (PMID: 25336721), which casts doubt on the identity of the cultured species.

UQ has only been reported outside *Pseudomonadota* in one abstract, specifically in the *Bacteroidia* species *Kaistella flava* (PMID: 33724915). The quinone profile (major quinone: MK_6 and "a few UQ_{10}") suggests a contamination likely originating from an alphaproteobacterium. The fortuitous presence of five homologs of the UQ pathway in a *Bacteroidota* genome led us to infer the presence of the UQ pathway in this phylum for the first time (S1 Table). However, the specificity of the annotations is complicated by the fact that some proteins in the quinone biosynthesis pathways belong to large families of proteins with various functions. The inference of the UQ pathway in a *Bacteroidota* genome was easily dismissed as an annotation error, since *Bacteroidota* homologs were localized in different parts of the genome rather than in compact genetic loci where *ubi* genes are usually found (Fig 3A) (2).

Text S2

The length of the polyprenyl tail of quinones varies between organisms. For example, UQ and MK in *Escherichia coli* have tails composed of 8 prenyl units and are therefore abbreviated UQ_8 and MK_8, whereas humans synthesize UQ_{10}, *Pseudomonas aeruginosa* has UQ_9, and *Bacillus subtilis* has MK_7. The length of the tail is determined by polyprenyl diphosphate synthases but remains difficult to predict from protein sequences and structures (3). The polyprenyl diphosphate synthases often do not exhibit exquisite selectivity, resulting in a major tail length *n* but also minor amounts of *n-1* and *n+1* isoprenologs, as documented for example in *Acetobacter aceti*, which contains UQ_9 as the major quinone, and UQ_8 and UQ_{10} as minor isoprenologs (4). The tail varies not only in length but also in degree of saturation. Indeed, the C=C double bond of one or more isoprenyl units may be reduced,

resulting in partially or fully saturated tails, which will decrease the flexibility of membranes (5). Gram-positive bacteria often contain MK with one or two saturations, designated $MK_n(H_2)$ and $MK_n(H_4)$, respectively (4), while several archaeal species belonging to the *Nitrososphaeria* (formerly Thaumarchaeota) or the *Desulfurococcales* contain a fully saturated MK_6 (designated $MK_{6:0}$, according to a different nomenclature) (6). Other alterations of the polyisoprenyl chain have seldomly been documented, as the presence of additional groups found in the chlorobiumquinone and the myxoquinone (7, 8) (Fig. S1).

Text S3

In several cases, the same species appears multiple times in the results (Table S2). This can result from several situations. Firstly, the same species may have been described in different articles. Secondly, species may have been renamed, resulting in multiple entries for what is now recognized as a single species in the NCBI taxonomy. Additionally, assignments based on the GTDB taxonomy can differ from those of the NCBI, reflecting updated or alternative taxonomic criteria. Thirdly, although most of the articles mentioned only one species, there are instances where several species are mentioned in the article title, particularly when taxonomic amendments are proposed (Fig. S8C-D). In such cases, the quinone descriptions found in the abstract are attributed to all species mentioned in the title.

318 species (based on the NCBI taxonomy) appear multiple times in the results. The quinone type is the same in all cases, except for *K. antarctica* and *K. jeonii.* Those two species were jointly reported in two distinct articles and were found to contain MK_6 in one case (PMID: 15653910) and MK_6 with minor quantities of UQ_{10} in the other (PMID: 33724915, see Text S1). For 254 out of 318 species (~80%), the reported chain lengths are the same. For 16 out of 318 species, the chain lengths cannot be compared because they are only specified in one abstract (Table S6). Of the 48 remaining cases displaying differences, 42 show partial overlap (Table S6). Most of these 48 cases correspond to species that are not the primary subject of the papers, but are mentioned in the context of taxonomic amendments, such as for *Kaistella* species (PMID: 33724915). For species (or even strains) that were described twice, for instance C*hakrabartia godavariana* (PMIDs: 31025129 and 31166165) and *Pseudarthrobacter phenanthrenivorans* (PMIDs: 31424381 and 19196765), the partial overlap in their reported quinone profiles appears to be due to the varying levels of details provided in each abstract. Some abstracts also report inconsistent information. For example, *Bhargavaea beijingensis* and *Bhargavaea ginsengi* were described as possessing either MK_7 (PMID: 19329597) or MK_8 (PMID: 22155760) (Tables S2 and S6). These examples illustrate the rare discrepancies in our data.

References

1. 2023. Biosynthesis and function of microbial methylmenaquinones, p. 1–58. *In* Advances in Microbial Physiology. Elsevier.

2. Chobert S-C, Roger-Margueritat M, Flandrin L, Berraies S, Lefèvre CT, Pelosi L, Junier I, Varoquaux N, Pierrel F, Abby SS. 2025. Dynamic quinone repertoire accompanied the diversification of energy metabolism in *Pseudomonadota*. The ISME Journal 19:wrae253.

3. Wallrapp FH, Pan J-J, Ramamoorthy G, Almonacid DE, Hillerich BS, Seidel R, Patskovsky Y, Babbitt PC, Almo SC, Jacobson MP, Poulter CD. 2013. Prediction of function for the polyprenyl transferase subgroup in the isoprenoid synthase superfamily. Proc Natl Acad Sci USA 110.

4. Collins MD, Jones D. 1981. Distribution of isoprenoid quinone structural types in bacteria and their taxonomic implication. Microbiol Rev 45:316–54.

5. Kellermann MY, Yoshinaga MY, Valentine RC, Wörmer L, Valentine DL. 2016. Important roles for membrane lipids in haloarchaeal bioenergetics. Biochim Biophys Acta 1858:2940–2956.

6. Elling FJ, Becker KW, Könneke M, Schröder JM, Kellermann MY, Thomm M, Hinrichs K. 2016. Respiratory quinones in *A rchaea* : phylogenetic distribution and application as biomarkers in the marine environment. Environmental Microbiology 18:692–707.

7. Panter F, Popoff A, Garcia R, Krug D, Müller R. 2022. Myxobacteria of the Cystobacterineae Suborder Are Producers of New Vitamin K2 Derived Myxoquinones. Microorganisms 10:534.

8. Powls R, Redfearn E, Trippett S. 1968. The structure of chlorobiumquinone. Biochemical and Biophysical Research Communications 33:408–411.
