# Supplementary material for: Global distribution of isoprenoid quinones across Bacteria
Source: mSystems. 2026 Jul 1;11(7):e01681-25. doi: 10.1128/msystems.01681-25 (PMC13386864; doi:10.1128/msystems.01681-25)
Supplement: Reviewer comments [file reviewer-comments.pdf]

AMERICAN SOCIETY FOR MICROBIOLOGY | mSystems®

Global distribution of isoprenoid quinones across Bacteria

Sophie-Carole Chobert, Suraj Kanwar, Olivier Lerouxel, Nelle Varoquaux, Julie Michaud, Ludovic Pelosi, Fabien Pierrel, and Sophie Abby

Corresponding Author(s): Sophie Abby, Centre National de la Recherche Scientifique

Review Timeline:

Submission Date:	November 28, 2025
Editorial Decision:	February 2, 2026
Revision Received:	April 10, 2026
Accepted:	May 5, 2026

Editor: Emile Gluck-Thaler

Reviewer(s): Disclosure of reviewer identity is with reference to reviewer comments included in decision letter(s). The following individuals involved in review of your submission have agreed to reveal their identity: Jing Wang (Reviewer #2)

Transaction Report:

(Note: With the exception of the correction of typographical or spelling errors that could be a source of ambiguity, letters and reports are not edited. The original formatting of letters and referee reports may not be reflected in this compilation.)

DOI: https://doi.org/10.1128/msystems.01681-25

Re: mSystems01681-25 (Global distribution of isoprenoid quinones across Bacteria)

Dear Dr. Sophie Saphia Abby:

Thank you for the privilege of reviewing your work. Below you will find my comments, instructions from the mSystems editorial office, and the reviewer comments.

The reviewer consensus is that this is a conceptually interesting study that provides an important new resource for the study of bacterial quinone evolution. Noted strengths of the manuscript include the combination of experimental physiological data with large scale comparative genomics, phylogenetics and text mining. The majority of reviewer comments concern how the results are presented and discussed, and several reviewers have important recommendations for strengthening the contextualization of the work within the current literature (including discussion of the Men and futalosine pathways and using quinones as chemotaxonomic markers), streamlining acronyms and improving the graphical representation of results. As well, there are concerns about the robustness of the bioinformatic results given the cutoffs used for determining pathway functionality. These concerns could be addressed through a sensitivity analysis or by including "and/or" operators into the search criteria. At minimum, you should consider presenting the results of multiple different cutoffs to convince the readers that the particular threshold that was chosen does not impact the main conclusions.

Please return the manuscript within 60 days; if you cannot complete the modification within this time period, please contact me. If you do not wish to modify the manuscript and prefer to submit it to another journal, notify me immediately so that the manuscript may be formally withdrawn from consideration by mSystems.

Revision Guidelines
To submit your modified manuscript, log into the submission site at https://msystems.msubmit.net/cgi-bin/main.plex. Go to Author Tasks and click the appropriate manuscript title to begin. The information you entered when you first submitted the paper will be displayed; update this as necessary. Note the following requirements:

• Upload point-by-point responses to the issues raised by the reviewers in a file named "Response to Reviewers," NOT in your cover letter.
• Upload a compare copy of the manuscript (without figures) as a "Marked-Up Manuscript" file.
• Upload a clean .DOC/.DOCX version of the revised manuscript and remove the previous version.
• Each figure must be uploaded as a separate, editable, high-resolution file (TIFF or EPS preferred), and any multipanel figures must be assembled into one file.
• Any supplemental material intended for posting by ASM should be uploaded with their legends separate from the main manuscript. You can combine all supplemental material into one file (preferred) or split it into a maximum of 10 files with all associated legends included.

For complete guidelines on revision requirements, see our Submission and Review Process webpage. Submission of a paper that does not conform to guidelines may delay acceptance of your manuscript.

Data availability: ASM policy requires that data be available to the public upon online posting of the article, so please verify all links to sequence records, if present, and make sure that each number retrieves the full record of the data. If a new accession number is not linked or a link is broken, provide mSystems production staff with the correct URL for the record. If the accession numbers for new data are not publicly accessible before the expected online posting of the article, publication may be delayed; please contact production staff (mSystems@asmusa.org) immediately with the expected release date.

Publication Fees: For information on publication fees and which article types are subject to charges, visit our website. If your manuscript is accepted for publication and any fees apply, you will be contacted separately about payment during the production process; please follow the instructions in that e-mail. Arrangements for payment must be made before your article is published.

ASM Membership: Corresponding authors may join or renew ASM membership to obtain discounts on publication fees. Need to upgrade your membership level? Please contact Customer Service at Service@asmusa.org.

The ASM Journals program strives for constant improvement in our submission and publication process. Please tell us how we can improve your experience by taking this quick Author Survey.

Thank you for submitting your paper to mSystems.

Sincerely,
Emile Gluck-Thaler
Editor
mSystems

Reviewer #1 (Comments for the Author):

The manuscript by Chobert et al. examines the distribution of membrane quinones among bacterial phyla. The last similar study was published 45 years ago. Therefore, the work by Chobert and her colleagues is very timely.

Comments

1. The manuscript links the function of quinones to ATP synthesis through the work of the ETC. Without questioning this statement, it should be noted that the functions of quinones are somewhat broader. Quinones are also found in anaerobic organisms incapable of respiration. In general, quinones participate in the exchange of electrons between redox-active components in the cytoplasm, cell membrane, and outside the cell. This exchange may not be coupled with energy conversion and ATP synthesis. Also quinones, through their long tails, seem to make membranes less permeable to ions. Hence, supporting respiratory electron transport associated with ATP synthesis is only one of many functions of quinones. In the revised manuscript, the function of quinones should be considered in a broader context.
2. The authors use traditional abbreviations to denote various quinones. A distinctive feature of this review is that it considers all quinones found in bacteria simultaneously. Therefore, using traditional abbreviations, each of which is suitable for describing a single quinone, leads to confusion when referring to all quinones at once. First, some quinones are designated by the letter "Q" (from "quinone"), while menaquinones are designated by the letter "K" (from "vitamin K"). Menaquinones could be designated as "MQ" without any loss of clarity. Another source of confusion is the similarity between the abbreviations for demethylmenaquinone (DMK) and dimethylmenaquinone (DMMK). DMK is a menaquinone that lacks its methyl group. DMMK, on the other hand, is a menaquinone that has two additional methyl groups. Perhaps DMK should be written as DeMK or DeMQ to emphasize the difference between "de" and "di"? It is also advisable to write DMMK and MMK separately, since these are two different quinones, rather than using the abbreviation (D)MMK. All of these suggestions are just recommendations.
3. Figure S1 should be moved to the main text. For each quinone structure, the abbreviation used in the work (if used) should be indicated in addition to the full name.
4. Fig. 1. There is a discrepancy between the figure and the caption. The colors mentioned in the caption-"very light blue," "very light yellow," and "very light green"-cannot be distinguished in the figure. The figure should be brought into line with the caption.
5. Line 219: Spaces should be inserted between the words.
6. Neither the Men pathway nor the futalosine pathway is described in the paper. There is also no discussion of how these two pathways differ from each other. Since the paper is intended for a general audience, information about these pathways should be included in the manuscript, especially since the futalosine pathway was "recently discovered". These points can be briefly addressed in the main text, and the complete schemes of the two pathways can be provided in the supplementary file.
7. Figure 2 should be presented as a zoomable image. Currently, it is impossible to read the names of the organisms.

Reviewer #2 (Comments for the Author):

Chobert et al. provide a comprehensive analysis of the "Global distribution of isoprenoid quinones across Bacteria" through genome annotation combined with literature mining, including experimental validation of a special case in Desulfobacterota. Given the ubiquity and crucial role of isoprenoid quinones in bioenergetics, this study provides fundamental data supporting future research in microbial physiology and biochemistry, genome-guided microbial cultivation, refined microbial ecological analysis (particularly for evaluating redox requirements of key microbial groups in environmental meta-omics data), co-evolution of microbes and Earth's environment, among others. The curated dataset also offers valuable resources for training and developing new analytical tools.

While the manuscript is clearly written overall, the introduction requires substantial revision to better highlight the core contributions of this work. The authors appear to have attempted to include all basic information about isoprenoid quinones, which, while educational, gives the manuscript a review-like quality that dilutes the logical framework of the research. The authors' previous paper (https://doi.org/10.1093/ismejo/wrae253) presents a much clearer introduction than the current manuscript.

Additionally, while the genome annotation method for predicting isoprenoid quinones was previously established by the authors, I do not see this as a limitation to the novelty of the current work. However, the authors may have concerns about this perception, which appears to have led them to position literature mining on equal footing with genome annotation. This emphasis on literature mining, while valuable, pushes the manuscript toward a review-style presentation and somewhat weakens its identity as a research article. In my view, literature mining primarily serves to validate genome annotation results and supplement information about saturation and chain length. Given the limited number and species coverage of existing literature, the significance of genome annotation deserves more prominent emphasis, especially considering that a major finding-the discovery of UQ presence in a Desulfobacterota class-was achieved through genome annotation. This discovery clearly demonstrates the power and value of the genome-based approach.

Specific Comments:

Introduction:

The introduction currently contains extensive general information about isoprenoid quinones, which, while educational, gives the manuscript a review-like attribute rather than that of a research article. This dilutes the main logical framework and important findings of the study. I would suggest rephrasing this section to better highlight the core contributions and discoveries of this

work. Additionally, the technical innovations and scientific highlights deserve more emphasis, as the current presentation might lead readers to perceive this as primarily a literature mining and compilation exercise rather than the significant research contribution it represents.
Line 97: The statement about something being "currently impossible" seems out of place in the introduction, particularly if this study doesn't aim to address this limitation. This content would be more appropriately placed in the discussion section.
Lines 96 and 112: There appear to be missing periods in these lines. Please review and correct these minor grammatical issues.
Lines 117-130: I would suggest citing recent studies that have also employed isoprenoid quinones as chemotaxonomic markers, such as https://doi.org/10.1128/AEM.02736-17 and https://doi.org/10.1111/1462-2920.13086. These references would strengthen the contextualization of your work within the current literature.
Results:
Lines 153-154: The relationship between "159 orders" and "91% containing UQ" needs clarification. Could you please specify whether this means 91% of orders (159 out of the total number) in Pseudomonadota contain UQ? Additionally, would "ubiquitous" be more appropriate than "diverse" in describing this widespread distribution pattern?
Lines 162-164: The requirements for RQ production need clarification. Does RQ production generally require the genetic potential for UQ production plus the RquA protein (as shown in Fig. 3E), or is it unusual for genomes to have genetic potential for both RQ and UQ production? Also, how does the fact that the GWC2-55-46 genome harbors genetic potential for UQ production support the prediction of its RQ production? This connection could be explained more explicitly.
Lines 209-210: Figure 1 contains only 751 representative genomes, which seems insufficient to support the claim of 98% consistency among 4,824 species. If this broader analysis is important to your narrative, I would recommend providing a supplementary table with the complete dataset to substantiate this claim.
Line 219: There appear to be missing spaces between words in this line. Please check and correct this formatting issue.
Line 274: The genome GCA_024255045.1 mentioned here doesn't appear to be shown in Fig. 3A. Could you please verify whether this should be GCA_024275045.1, as shown in the figure?
Lines 284-285: Please clarify that the increased UQ production is compared to the empty plasmid condition (presumably with the corresponding host gene knocked out) rather than to wild-type conditions. This distinction is important for proper interpretation of the results.
Discussion:
Just a soft recommendation that doesn't necessarily need to be addressed: the discussion would benefit from addressing archaeal quinone systems, or at least including some commentary on this topic. The ubiquity of saturated menaquinones in Archaea compared to Bacteria suggests that these compounds may represent ancestral and diagnostic features of the domain. Furthermore, overlaps between quinone compositions of distinct thermophilic and halophilic archaea and bacteria might indicate lateral gene transfer events. Including this broader phylogenetic perspective would enhance the comprehensiveness of this study.
Figures:
Figure 1: The pink dots appear to be present only on branches lacking color coding, which suggests that incongruence between literature mining and genome annotation occurs only when quinone production is experimentally confirmed but not genetically annotated. However, this interpretation seems inconsistent with the description in the Results section (e.g., lines 210-213). Could you please clarify this apparent discrepancy? Additionally, the figure legend mentions "very light blue" and "very light green" for unpredicted quinone pathways, but these colors appear to be absent from the figure, with gray being used instead. Please update the legend to accurately reflect the color scheme used in the figure.
Figure 3: As mentioned earlier, genome GCA_024255045.1 (referenced in Line 274) doesn't appear in Fig. 3A. Please verify whether this should be GCA_024275045.1 as shown in the figure.
Methods:
Lines 473-476: The use of minimum detected gene numbers as a cutoff for determining pathway functionality raises some concerns. Given that genes can have various relationships (upstream/downstream interactions, different subunits of the same protein complex, or functional redundancy), this approach may not adequately reflect a pathway's functional potential. Have you considered adopting a more sophisticated approach, such as the logic-based system used by KEGG pathways that employs brackets, "and," and "or" operators? Such an approach might provide a more nuanced and accurate assessment of pathway functionality.

Reviewer #3 (Comments for the Author):

Chorbert et al presented a study of isoprenoid quinone biosynthetic pathways mined from across 26,264 bacterial genomes from GTDB and from the literature. The work reveals novel insights into quinone pathway evolution, particularly the discovery and experimental validation of an O_2-independent ubiquinone (UQ) biosynthetic pathway in the class GWC2-55-46 (Desulfobacterota). In my opinion, the integration of comparative genomics, phylogenomics, and targeted heterologous complementation experiments is methodologically sound. In my opinion, the paper is well written, appropriately scoped for mSystems, and the data resource will be valuable to the microbiology community.

Below are some feedback that, I hope, may improve the work.

• The authors may consider a sensitivity analysis to show how conclusions change if thresholds are varied (e.g., {plus minus}1 gene). Particularly for the claim of "lineages devoid of quinone pathways," this is important for confidence.

• For the GWC2-55-46 class, the authors state that MAGs are "high quality" and reference GTDB criteria but do not explicitly report genome completeness or contamination estimates. Please add these metrics (e.g., from CheckM, CheckM2 or BUSCO) for all nine MAGs in a supplementary table.
• Regarding claims and observations for the MK pathway evolution, where authors suggest lateral gene transfer as the mechanism that may explain the apparent distribution of the MK pathway:
○ the author could perhaps support this claim by simulating LGT and comparing the resulting tree to the observed one.
○ Or perform gene-tree/species-tree reconciliation or ancestral state reconstruction (ASR)

• According to GTDB db stats (https://gtdb.ecogenomic.org/stats/r220), there are 27,292 high quality representative genomes. My question is why did you only download 26,264?
• L219: no spacing
L294: Cyanobacteriia - Cyanobacteria

Reviewers' comments

Editor's comments:

The reviewer consensus is that this is a conceptually interesting study that provides an important new resource for the study of bacterial quinone evolution. Noted strengths of the manuscript include the combination of experimental physiological data with large scale comparative genomics, phylogenetics and text mining. The majority of reviewer comments concern how the results are presented and discussed, and several reviewers have important recommendations for strengthening the contextualization of the work within the current literature (including discussion of the Men and futalosine pathways and using quinones as chemotaxonomic markers), streamlining acronyms and improving the graphical representation of results. As well, there are concerns about the robustness of the bioinformatic results given the cutoffs used for determining pathway functionality. These concerns could be addressed through a sensitivity analysis or by including "and/or" operators into the search criteria. At minimum, you should consider presenting the results of multiple different cutoffs to convince the readers that the particular threshold that was chosen does not impact the main conclusions.

We thank the editor and the three reviewers for the positive assessment of our work and for the constructive criticisms that helped to significantly improve our manuscript, in particular by pushing us to make more evident the original contributions and discoveries brought by the genomic annotation part and remodelling the Introduction, abstract and importance statement. We have also addressed the concerns about the robustness of the bioinformatic analyses by explaining our choices and demonstrating that they do not affect the major conclusions of the article. We have taken into account the comments and provide detailed response to all of them in the following.

Reviewer #1 (Comments for the Author):

The manuscript by Chobert et al. examines the distribution of membrane quinones among bacterial phyla. The last similar study was published 45 years ago. Therefore, the work by Chobert and her colleagues is very timely.

Comments

1. The manuscript links the function of quinones to ATP synthesis through the work of the ETC. Without questioning this statement, it should be noted that the functions of quinones are somewhat broader. Quinones are also found in anaerobic organisms incapable of respiration. In general, quinones participate in the exchange of electrons between redox-active components in the cytoplasm, cell membrane, and outside the cell. This exchange may not be coupled with energy conversion and ATP synthesis. Also quinones, through their long tails, seem to make membranes less permeable to ions. Hence, supporting respiratory electron transport associated with ATP synthesis

is only one of many functions of quinones. In the revised manuscript, the function of quinones should be considered in a broader context.

We agree that quinones have numerous important physiological roles beyond their involvement in ETC. In fact, these roles were already discussed at length in Introduction on lines 56-66 “Besides this well-established role…” where we give an overview of their involvement in other cellular processes and pathways, and their potential role in membrane fluidity and metabolic shifts.

2. The authors use traditional abbreviations to denote various quinones. A distinctive feature of this review is that it considers all quinones found in bacteria simultaneously. Therefore, using traditional abbreviations, each of which is suitable for describing a single quinone, leads to confusion when referring to all quinones at once. First, some quinones are designated by the letter "Q" (from "quinone"), while menaquinones are designated by the letter "K" (from "vitamin K"). Menaquinones could be designated as "MQ" without any loss of clarity. Another source of confusion is the similarity between the abbreviations for demethylmenaquinone (DMK) and dimethylmenaquinone (DMMK). DMK is a menaquinone that lacks its methyl group. DMMK, on the other hand, is a menaquinone that has two additional methyl groups. Perhaps DMK should be written as DeMK or DeMQ to emphasize the difference between "de" and "di"? It is also advisable to write DMMK and MMK separately, since these are two different quinones, rather than using the abbreviation (D)MMK. All of these suggestions are just recommendations.

We agree with this reviewer that the abbreviations are plenty and can be confusing at once, but these are already well established and adopted in the literature, and became “tradition”. This is why we would rather not deviate too much from this well-established nomenclature and keep menaquinone as “MK” as this if far more used than “MQ” from an analysis of PubMed results. Also, based on this reviewer suggestion, we propose the following abbreviations, introduced in the Introduction:

- DMK for demethylmenaquinone
- MMK for methylmenaquinone
- diMMK for dimethylmenaquinone

And we now clearly dissociate in the text the methylmenaquinone and the dimethylmenaquinone by suppressing the confusing (D)MMK abbreviation. The abbreviations of the quinones discussed in this study are introduced on Fig. 1 that was amended to reflect this change (see also reply to the next comment).

3. Figure S1 should be moved to the main text. For each quinone structure, the abbreviation used in the work (if used) should be indicated in addition to the full name.

Fig S1 introduces the structure of many more quinones that we can detect with the genomic and text mining approach, and the structure of the main quinones discussed in the text are already presented on Figure 1 along with their abbreviations (see also above), hence we decided to leave Fig. S1 as a supplementary figure and updated Fig 1 to introduce the new abbreviations and present the structure of the MMK.

4. Fig. 1. There is a discrepancy between the figure and the caption. The colors mentioned in the caption-"very light blue," "very light yellow," and "very light green"-cannot be distinguished in the figure. The figure should be brought into line with the

caption.
Thank you for pointing this, this was done: the caption of the figure was amended to reflect the actual colours on the figure.

5. Line 219: Spaces should be inserted between the words.
Thank you for pointing this, this was done.

6. Neither the Men pathway nor the futalosine pathway is described in the paper. There is also no discussion of how these two pathways differ from each other. Since the paper is intended for a general audience, information about these pathways should be included in the manuscript, especially since the futalosine pathway was "recently discovered". These points can be briefly addressed in the main text, and the complete schemes of the two pathways can be provided in the supplementary file.

We thank the reviewer for raising this point. Indeed, both pathways were not detailed, and this information is important especially since we make a focus on shifts between the pathways… We have now remodelled the Introduction to make a stronger focus about the state-of-the-art on the quinone pathways, we have introduced a text about the two MK pathways, and do provide a novel supplementary figure that shows them (new Fig. S2).

7. Figure 2 should be presented as a zoomable image. Currently, it is impossible to read the names of the organisms.
We agree that this was needed, we have now uploaded a high-quality PDF for Figure S2 (now Fig. S3). Please also note that the now Fig. S3 presents only a portion of the underlying genome annotation data. All information on the ~26,000 annotated genomes can be consulted in the Supplementary Table S1. In the same spirit, we now also provide a labelled, high-quality version of the tree presented in Fig. 2C in Fig. S4.

Reviewer #2 (Comments for the Author):

Chobert et al. provide a comprehensive analysis of the "Global distribution of isoprenoid quinones across Bacteria" through genome annotation combined with literature mining, including experimental validation of a special case in Desulfobacterota. Given the ubiquity and crucial role of isoprenoid quinones in bioenergetics, this study provides fundamental data supporting future research in microbial physiology and biochemistry, genome-guided microbial cultivation, refined microbial ecological analysis (particularly for evaluating redox requirements of key microbial groups in environmental meta-omics data), co-evolution of microbes and Earth's environment, among others. The curated dataset also offers valuable resources for training and developing new analytical tools.
While the manuscript is clearly written overall, the introduction requires substantial revision to better highlight the core contributions of this work. The authors appear to have attempted to include all basic information about isoprenoid quinones, which, while educational, gives the manuscript a review-like quality that dilutes the logical framework of the research. The authors' previous paper (https://doi.org/10.1093/ismejo/wrae253) presents a much clearer introduction than the current manuscript.

Additionally, while the genome annotation method for predicting isoprenoid quinones

was previously established by the authors, I do not see this as a limitation to the novelty of the current work. However, the authors may have concerns about this perception, which appears to have led them to position literature mining on equal footing with genome annotation. This emphasis on literature mining, while valuable, pushes the manuscript toward a review-style presentation and somewhat weakens its identity as a research article. In my view, literature mining primarily serves to validate genome annotation results and supplement information about saturation and chain length. Given the limited number and species coverage of existing literature, the significance of genome annotation deserves more prominent emphasis, especially considering that a major finding-the discovery of UQ presence in a Desulfobacterota class-was achieved through genome annotation. This discovery clearly demonstrates the power and value of the genome-based approach.

We thank this reviewer for the very positive assessment of our work and these valuable constructive suggestions. We provide now a completely remodelled Introduction that better introduces the original contributions and discoveries provided by the genome annotation approach in this work (more details below). We also remodelled the abstract and Importance statement accordingly.

Specific Comments:

Introduction:

The introduction currently contains extensive general information about isoprenoid quinones, which, while educational, gives the manuscript a review-like attribute rather than that of a research article. This dilutes the main logical framework and important findings of the study. I would suggest rephrasing this section to better highlight the core contributions and discoveries of this work. Additionally, the technical innovations and scientific highlights deserve more emphasis, as the current presentation might lead readers to perceive this as primarily a literature mining and compilation exercise rather than the significant research contribution it represents.

We totally agree with the reviewer. We thus remodelled the Introduction to take these comments into account, by:

- down-tuning the general information on quinone chain variations, which was transferred as a new Supplementary Text (now Sup Text S2).
- providing more information on the quinone pathways (include a new Fig. S2), and the current ability to now annotate them through genomic approach
- emphasizing better the novel discoveries

We also slightly modified the abstract and importance statement to reflect this change of perspective, where now genomic annotation is better introduced as a source of discovery.

Line 97: The statement about something being "currently impossible" seems out of place in the introduction, particularly if this study doesn't aim to address this limitation. This content would be more appropriately placed in the discussion section.
This statement has disappeared from the Introduction during its remodelling.

Lines 96 and 112: There appear to be missing periods in these lines. Please review and correct these minor grammatical issues.

Thank you, this was done.

Lines 117-130: I would suggest citing recent studies that have also employed isoprenoid quinones as chemotaxonomic markers, such as https://doi.org/10.1128/AEM.02736-17 and https://doi.org/10.1111/1462-2920.13086. These references would strengthen the contextualization of your work within the current literature.
Thanks for the suggestion, we indeed know these studies well and really value them. However, we could not find a suitable place to cite them after remodelling the Introduction.

Results:
Lines 153-154: The relationship between "159 orders" and "91% containing UQ" needs clarification. Could you please specify whether this means 91% of orders (159 out of the total number) in Pseudomonadota contain UQ? Additionally, would "ubiquitous" be more appropriate than "diverse" in describing this widespread distribution pattern?
We agree this sentence was unclear, we have now revised it. The 91% (which was actually fixed to 92% as it was exactly 91.8%) referred indeed to the orders, and the “diversity” referred to the Pseudomonadota lineage itself, not the pattern of UQ presence, which is indeed very largely distributed.

Lines 162-164: The requirements for RQ production need clarification. Does RQ production generally require the genetic potential for UQ production plus the RquA protein (as shown in Fig. 3E), or is it unusual for genomes to have genetic potential for both RQ and UQ production? Also, how does the fact that the GWC2-55-46 genome harbors genetic potential for UQ production support the prediction of its RQ production? This connection could be explained more explicitly.

We agree and while remodelling the Introduction, we have now clarified that RQ is obtained in one step with RquA from UQ, hence RQ production requires both the UQ pathway and RquA: “In Pseudomonadota, the low potential quinone RQ was shown to be produced in one step from UQ through the action of the RquA protein”.

Lines 209-210: Figure 1 contains only 751 representative genomes, which seems insufficient to support the claim of 98% consistency among 4,824 species. If this broader analysis is important to your narrative, I would recommend providing a supplementary table with the complete dataset to substantiate this claim.
This study of consistency was made by cross-referencing Tables S1 and S2, not only from the data presented in Fig. 1. To make this more evident and traceable to the reader, we have now added two columns in Table S2 indicating the consistency between genomic annotations and the text mining results: “genomic_correspondence” and “textmining_genome_consistency”. The “textmining_genome_consistency” column contains three possible values: consistent, inconsistent, and incomparable.
- “Consistent” is assigned when genomic annotations and text mining data were the same.
- “Incomparable” is assigned when the organism produces a quinone for which the biosynthetic pathway cannot be predicted (e.g., methylene-UQ or DMMK) or when no corresponding genome was available.

- “Inconsistent” is assigned when there is at least one discrepancy between the genomic annotation and text-mining data (e.g., DMK identified solely via genomic annotation, but DMK + MK detected through text-mining)."
We have also added a small section in the Material and Methods section about the comparison of genome annotation and text mining data “Comparison of genomic annotations and text mining data”, explaining how it was done precisely.

Indeed, when the results could be compared, that is, across 4,853 unique species, 98% of the genomic annotations were consistent with the text mining results.

Line 219: There appear to be missing spaces between words in this line. Please check and correct this formatting issue.
Thank you, this was done.

Line 274: The genome GCA_024255045.1 mentioned here doesn't appear to be shown in Fig. 3A. Could you please verify whether this should be GCA_024275045.1, as shown in the figure?
Thanks a lot for pointing this, this was indeed a mistake! We have corrected this.

Lines 284-285: Please clarify that the increased UQ production is compared to the empty plasmid condition (presumably with the corresponding host gene knocked out) rather than to wild-type conditions. This distinction is important for proper interpretation of the results.
Thank you, this was indeed missing and was done.

Discussion:
Just a soft recommendation that doesn't necessarily need to be addressed: the discussion would benefit from addressing archaeal quinone systems, or at least including some commentary on this topic. The ubiquity of saturated menaquinones in Archaea compared to Bacteria suggests that these compounds may represent ancestral and diagnostic features of the domain. Furthermore, overlaps between quinone compositions of distinct thermophilic and halophilic archaea and bacteria might indicate lateral gene transfer events. Including this broader phylogenetic perspective would enhance the comprehensiveness of this study.

We agree that this is an interesting point to raise in Discussion. We have now included it as a final section, that indeed enables more comprehensiveness to this study, and provides an opening towards future investigations.

Figures:
Figure 1: The pink dots appear to be present only on branches lacking color coding, which suggests that incongruence between literature mining and genome annotation occurs only when quinone production is experimentally confirmed but not genetically annotated. However, this interpretation seems inconsistent with the description in the Results section (e.g., lines 210-213). Could you please clarify this apparent discrepancy? Additionally, the figure legend mentions "very light blue" and "very light green" for unpredicted quinone pathways, but these colors appear to be absent from the figure, with gray being used instead. Please update the legend to accurately reflect the color scheme used in the figure.

Well, pink dots indeed do convey the fact that the text mining recovered the presence of a quinone whose pathway was not detected using the genome annotation as indicated in the legend. The converse would be quite difficult to obtain (pathway annotated but no corresponding quinone found in quinone detection assay), as we have selected abstracts based on the mention of detected quinones. We believe that the main text is well aligned with the depiction of the results in Fig. 1 even though it does not contain all analysed data. For instance, all four cases where “MK was measured in four species, none of which had a MK pathway in their genome” are represented in Figure 1 with the corresponding pink dots. Table S2 now provides more details regarding the comparison of text mining versus genome annotation.
As for the text legend, it was indeed off regarding the colours, we corrected it.

Figure 3: As mentioned earlier, genome GCA_024255045.1 (referenced in Line 274) doesn't appear in Fig. 3A. Please verify whether this should be GCA_024275045.1 as shown in the figure.
Yes, you are right, this was indeed a typo in the main text, this was corrected.

Methods:
Lines 473-476: The use of minimum detected gene numbers as a cutoff for determining pathway functionality raises some concerns. Given that genes can have various relationships (upstream/downstream interactions, different subunits of the same protein complex, or functional redundancy), this approach may not adequately reflect a pathway's functional potential. Have you considered adopting a more sophisticated approach, such as the logic-based system used by KEGG pathways that employs brackets, "and," and "or" operators? Such an approach might provide a more nuanced and accurate assessment of pathway functionality.

The choices for the minimal detected genes stem from our recent studies, as we actually recently provided data supporting the chosen threshold in Fig S8 from this article: https://academic.oup.com/ismej/article/19/1/wrae253/7927901.
In addition to the data brought by our recent studies, we have evaluated to 154 extra genomes that will obtain an annotation for MK if we would drop the number of minimum genes required from 6 to 5. Therefore, this would not change the major conclusions we draw for the global distribution of the quinone pathways. In particular, of the 26,264 genomes analysed, there will still be 3,399 without any predicted quinone pathway. These results are presented in a new sheet 3 in Table S1 which is now referred to in the Results section, with a sentence about the impact of lowering the cutoff. For the UQ pathway, as we have now a quite extensive view of its variations from both computational and experimental studies (https://doi.org/10.1093/molbev/msad219) and made thorough investigations about its annotation, we believe our approach is very robust as is. We already dedicated a quite thorough discussion on the matter in the Discussion section of this article: https://academic.oup.com/ismej/article/19/1/wrae253/7927901.
In order to clarify: there is no such thing as different subunits (i.e. each protein “stands” by itself) nor functional redundancy in these pathways – which make them rather straight-forward to annotate with our approach, except for the hydroxylases from the UQ pathway – which evolution and diversity was previously thoroughly investigated by us in this article https://doi.org/10.1093/molbev/msad219. This is why the “rules” for the UQ pathways are slightly more complicated than a sole threshold for the number

of proteins. This should also be clarified as we have now provided more information about UQ biosynthetic pathways in the Introduction.

Reviewer #3 (Comments for the Author):

Chorbert et al presented a study of isoprenoid quinone biosynthetic pathways mined from across 26,264 bacterial genomes from GTDB and from the literature. The work reveals novel insights into quinone pathway evolution, particularly the discovery and experimental validation of an O_2-independent ubiquinone (UQ) biosynthetic pathway in the class GWC2-55-46 (Desulfobacterota). In my opinion, the integration of comparative genomics, phylogenomics, and targeted heterologous complementation experiments is methodologically sound. In my opinion, the paper is well written, appropriately scoped for mSystems, and the data resource will be valuable to the microbiology community.

We thank this reviewer for their positive assessment of our work.

Below are some feedback that, I hope, may improve the work.

• The authors may consider a sensitivity analysis to show how conclusions change if thresholds are varied (e.g., {plus minus}1 gene). Particularly for the claim of "lineages devoid of quinone pathways," this is important for confidence.

We thank the reviewer for raising this point. We have addressed it in a comment by another reviewer above. Here is our reply:

The choices for the minimal detected genes stem from our recent studies, as we actually recently provided data supporting the chosen threshold in Fig S8 from this article: https://academic.oup.com/ismej/article/19/1/wrae253/7927901.
In addition to the data brought by our recent studies, we have evaluated to 154 extra genomes that will obtain an annotation for MK if we would drop the number of minimum genes required from 7 to 6. Therefore, this would not change the major conclusions we draw for the global distribution of the quinone pathways. In particular, of the 26,264 genomes analysed, there will still be 3,399 without any predicted quinone pathway. These results are presented in a new sheet 3 in Table S1 which is now referred to in the Results section, with a sentence about the impact of lowering the cutoff. For the UQ pathway, as we have now a quite extensive view of its variations from both computational and experimental studies (https://doi.org/10.1093/molbev/msad219) and made thorough investigations about its annotation, we believe our approach is very robust as is. We already dedicated a quite thorough discussion on the matter in the Discussion section of this article: https://academic.oup.com/ismej/article/19/1/wrae253/7927901.

• For the GWC2-55-46 class, the authors state that MAGs are "high quality" and reference GTDB criteria but do not explicitly report genome completeness or contamination estimates. Please add these metrics (e.g., from CheckM, CheckM2 or BUSCO) for all nine MAGs in a supplementary table.
All annotated genomes indeed belong to the “high quality” subset from the GTDB database, including the MAGs from the GWC2-55-46 class. Quality information from CheckM2 is already presented for all genomes analysed in the study in the

supplementary Table S1 (sheet 2). We have renamed the corresponding columns to clarify this point.

• Regarding claims and observations for the MK pathway evolution, where authors suggest lateral gene transfer as the mechanism that may explain the apparent distribution of the MK pathway:

○ the author could perhaps support this claim by simulating LGT and comparing the resulting tree to the observed one.

○ Or perform gene-tree/species-tree reconciliation or ancestral state reconstruction (ASR)

We thank the reviewer for these suggestions. But we believe that the proposed analyses are difficult to implement given that we are dealing with a multi-gene trait, they would deserve a study by themselves and they currently fall beyond the scope of our analysis: our main point here was to highlight the highly dynamic behaviour of these pathways, not to conclude on the mechanisms. We can however add that in a recent study where the main focus was the dynamic behaviour of quinone pathways in *Pseudomonadota*, we demonstrated several occurrences of lateral gene transfer of the Men pathway : https://academic.oup.com/ismej/article/19/1/wrae253/7927901. This is why we suggested this mechanism as a potential one.
We tuned down the claim about the mechanism by removing "strongly" (from "strongly suggest") in the sentence in Results: "This pattern of switching between pathways suggests regular acquisition of one MK pathway by lateral gene transfer alongside the loss of the ancestral pathway", and added a note about the recent discovery of multiple cases of Men lateral gene transfer in Pseudomonadota, in the dedicated Discussion section.

• According to GTDB db stats (https://gtdb.ecogenomic.org/stats/r220), there are 27,292 high quality representative genomes. My question is why did you only download 26,264?
As bacteria were the main focus of our study, we indeed excluded archaeal genomes, hence explaining this difference.

• L219: no spacing
Thanks, this was done.

• L294: Cyanobacteriia – Cyanobacteria
Thanks for pointing this. But as weird as this may seem, this is not a typo but the current official name for the phylum.

Re: mSystems01681-25R1 (Global distribution of isoprenoid quinones across Bacteria)

Dear Dr. Sophie Abby:

Your manuscript has been accepted, and I am forwarding it to the ASM production staff for publication. Your paper will first be checked to make sure all elements meet the technical requirements. ASM staff will contact you if anything needs to be revised before copyediting and production can begin. Otherwise, you will be notified when your proofs are ready to be viewed.

Data Availability: ASM policy requires that data be available to the public upon online posting of the article, so please verify all links to sequence records, if present, and make sure that each number retrieves the full record of the data. If a new accession number is not linked or a link is broken, provide production staff with the correct URL for the record. If the accession numbers for new data are not publicly accessible before the expected online posting of the article, publication may be delayed; please contact ASM production staff immediately with the expected release date.

All custom bioinformatic scripts and algorithms must be uploaded to a publicly available data repository eg figshare with a DOI. Please also upload all phylogenetic tree files and sequence alignments, as these are necessary to replicate analysis

Publication Fees: For information on publication fees and which article types have charges, please visit our website. We have partnered with Copyright Clearance Center (CCC) to collect author charges. If fees apply to your paper, you will receive a message from no-reply@copyright.com with further instructions. For questions related to paying charges through RightsLink, please contact CCC at ASM_Support@copyright.com or toll free at +1-877-622-5543. CCC makes every attempt to respond to all emails within 24 hours.

ASM Membership: Corresponding authors may join or renew ASM membership to obtain discounts on publication fees. Need to upgrade your membership level? Please contact Customer Service at Service@asmusa.org.

Embargo Policy: A press release may be issued as soon as the manuscript is posted on the mSystems Latest Articles webpage. The corresponding author will receive an email with the subject line "ASM Journals Author Services Notification" when the article is available online.

Cover Image Submissions: If you would like to submit a potential Cover Image, please email a file and a short legend to msystems@asmusa.org. Please note that we can only consider images that (i) the authors created or own and (ii) have not been previously published. By submitting, you agree that the image can be used under the same terms as the published article. Image File requirements: TIF/EPS, 7.5 inches wide by 8.25 inches tall (at least 2,250 pixels wide by 2,475 pixels tall), minimum 300 dpi resolution (600 dpi preferred), RGB, and no figure elements, e.g., arrows or panel labels. The legend should be a short description of the image, 1-2 sentences recommended. Please download and use this interactive template in Adobe to ensure that your proposed cover image meets our size requirements (https://journals.asm.org/pb-assets/pdf-text-excel-files/ASM-Interactive-Sizing-Cover-Template-1715689791.pdf).

Thank you for submitting your paper to mSystems.

Sincerely,
Emile Gluck-Thaler
Editor
mSystems

Reviewer #1 (Comments for the Author):

The manuscript can be published as it is.

Reviewer #2 (Comments for the Author):

All concerns and issues have been addressed. I have no further questions.
